# Association between Metabolic Syndrome and Chronic Kidney Disease in Perimenopausal Women

**DOI:** 10.3390/ijerph10093987

**Published:** 2013-08-30

**Authors:** Yongqiang Li, Liqin Zhao, Youming Chen, Aiqun Liu, Xinyu Liu, Xiaofei Shao, Ying Zhang, Honglei Wang, Xiaohong Wang, Bin Li, Kangping Deng, Qin Liu, Harry Holthöfer, Hequn Zou

**Affiliations:** 1 Department of Nephronology, Institute of Nephronology and Urology, The Third Affiliated Hospital of Southern Medical University, Guangzhou 510630, China; E-Mails: liyongqiang851@163.com (Y.L.); nanyitx@126.com (A.L.); pepsi84@163.com (X.L.); shaoxfei@126.com (X.S.); xiaoying2001-205@163.com (Y.Z.); ailemonmint@126.com (H.W.); liyongqiang851@126.com (X.W.); hhhlibin@126.com (B.L.); kangpingdeng82@163.com (K.D.); aiq2006@126.com (Q.L.); 2 The Department of Obstetrics and Gynecology, The Third Affiliated Hospital of Southern Medical University, Guangzhou 510630, China; E-Mail: liyongqiang851@sohu.com; 3 Clinical laboratory, the Third Affiliated Hospital of Southern, Medical University, Guangzhou 510630, China; E-Mail: cym38432@163.com; 4 National Centre for Sensor Research/BioAnalytical Sciences, Dublin City University, Dublin 9, Ireland; E-Mail: harry.holthofer@dcu.ie

**Keywords:** metabolic syndrome, chronic kidney disease, perimenopausal women

## Abstract

The purpose of the study was to explore the association between metabolic syndrome (MetS) and chronic kidney disease (CKD) in perimenopausal women. A cross-sectional study was conducted in Zhuhai from June to October 2012. Perimenopausal women (n = 685) were included in the study. All participants were divided into three subgroups: Group 1, 40 years old ≤ Age < 50 years old; Group 2, 50 years old ≤ Age < 60 years old; Group 3, 60 years old ≤ Age ≤ 65 years old. MetS was associated with CKD (*p* < 0.01) in the unadjusted analyses in total subjects. After adjusting the potential confounders, the odd ratios of CKD for MetS was 2.66 (95% CI 1.56 to 4.49, *p* < 0.001). There was no relationship between MetS and CKD in both Group 1 and Group 3. MetS was associated with CKD (*p* < 0.001) in the unadjusted analyses in Group 2. After adjusting for potential confounders, MetS was significantly associated with CKD. The odd ratios for MetS was 6.79 (95% CI 2.30 to 20.09, *p* < 0.001). There was no relationship between elevated blood pressure, elevated fasting glucose, abdominal obesity, Low HDL cholesterol, elevated triglycerides and CKD in both Group 1 and Group 3. Elevated blood pressure was associated with CKD in Group 2 (unadjusted Odds ratio: 4.52 (1.28–16.02), *p* = 0.02). After adjusting for potential confounders, there was no relationship between elevated blood pressure and CKD (*p* = 0.78). Elevated fasting glucose was associated with CKD in Group 2 (unadjusted Odds ratio: 3.69 (1.10–12.38), *p* = 0.03). After adjusting for potential confounders, there was no relationship between elevated fasting glucose and CKD (*p* = 0.15). There was no relationship between abdominal obesity, Low HDL cholesterol, elevated triglycerides and CKD in Group 2. These findings suggest that in perimenopausal women aged from 50 or older to 60 MetS was associated with CKD. There is no relationship between MetS and CKD in perimenopausal women aged from 40 or older to 50 and aged from 60 or older to 65.

## 1. Introduction

Despite recent advances in the treatment of chronic kidney disease (CKD) [1,2,3], it remains an important public health challenge [4,5]. A national survey of chronic kidney disease conducted by Zhang *et al*. [6] established the overall prevalence of CKD in China to be 10.8% in 2012. CKD is a major risk factor for cardiovascular disease (CVD) and premature death [7,8,9]. More and more studies have begun to focus on the pre-CKD stage [10,11]. Metabolic syndrome (MetS), characterized by abdominal obesity, hypertriglyceridemia, depressed serum high-density lipoprotein cholesterol (HDL-C), hypertension and diabetes [12], is commonly found in patients afflicted with CKD. MetS and it’s components have been associated with a subsequent progression to CKD [13]. The term perimenopause should include the period immediately prior to menopause (when the endocrinological, biological and clinical features of approaching menopause commence) and the five years after menopause. Although it is a physiological feature of every woman’s lifespan, it is often associated with numerous metabolic changes that determine the personal well-being and general health of a woman [14]. The prevalence of MetS increases during the premenopausal through the postmenopausal years of women, independent of age [15]. Perimenopausal women are have higher risks of developing CKD [16].

To the best of our kowledge, relatively few studies have focused on the relationship between MetS and CKD in perimenopausal women, so this study was designed to clarify the association between MetS and the risk of CKD by initiating this door-to-door community cross-sectional screening study of the perimenopausal women population of Wanzhai Town, ZhuHai city, one of the leading commercial cities in Southern China.

## 2. Methods

### 2.1. Participants

This cross-sectional study was conducted in Wanzhai Town, Zhuhai City, a prominent commercial city in Southern China. Data was collected from 763 community perimenopausal women residents, 40–65 years of age from June to October, 2012. Of these, 685 community perimenopausal women met the inclusion criteria. Participants were recruited by mail and home visits. All perimenopausal women community residents gave their written informed consent. This study was approved by The Ethics Committee of The Third Affiliated Hospital of Southern Medical University, Guangzhou. This study was performed fulfilling the principles of Helsinki Declaration and the International Guidelines for Ethical Review for Epidemiological Studies.

### 2.2. Study Measurables

Sociodemographic characteristics, including personal health history (coronary artery disease, stroke, hypertension, and diabetes) and details about lifestyle (smoking status, alcohol intake, diet habits and physical activity) were obtained by questionnaire. Body weight, height, waist circumference and blood pressure were measured with standardized protocol in the morning between 08:00 and 11:00 am. Central obesity was defined on the basis of ethnic specific values of waist circumference [17]. Blood pressure was measured twice to the nearest 2 mmHg by a trained nurse using a mercury totally closed desk-top sphygmomanometer (Model XJ300/40-1, Shanghai, China), after the participants had been seated at least 5 min. The first and fourth Korotkoff sounds were used to represent the systolic and diastolic blood pressure. The average value of these two measuring points for systolic and diastolic blood pressure was recorded.

Blood glucose level was measured with a hexokinase enzyme reference method and serum creatinine (SCr) with an enzymatic method on an autoanalyzer (Hitachi 7170, Hitachi, Tokyo, Japan). Serum high-density lipoprotein (HDL) was determined enzymatically with commercially available reagents (Shanghai Gensource Co., Ltd, Shanghai, China), and cholesterol and triglyceride (TG) levels were determined enzymatically with commercially available reagents (Roche Diagnostics, Mannheim, Germany). Low density lipoprotein cholesterol(LDL-C) was measured by the Friedewald formula. High sensitivity C-reactive protein was measured by enzymatic Turbidimetric immunoassay method (Orion Diagnostica Oy, Espoo, FinLand). Urinary albumin and creatinine were measured from a fresh morning spot urine sample or first morning urine sample stored at +4 °C for less than 1 week. Albuminuria was measured with immunoturbidimetric tests (Audit Diagnostics, Cork, Ireland). Urinary creatinine was measured with Jaffe’s kinetic method [18]. The urinary albumin to creatinine ratio (ACR; mg/g creatinine) was calculated.

### 2.3. Determination of Metabolic Syndrome

There are different criteria to define MetS [19,20]. In our study, according to the definition of the International Diabetes Federation (IDF) [21] MetS can be diagnosed when central obesity (waist circumference >90 cm for men or >80 cm for women) is accompanied by any two of the following four factors: (1) a TG level of 1.7 mmol/L or greater; (2) an HDL cholesterol level lower than 1.03 mmol/L for men or lower than 1.29 mmol/L for women; (3) a blood pressure (BP) of 130/85 mmHg or higher or receiving treatment of previously diagnosed hypertension; (4) a fasting blood glucose (FBG) of 5.6 mmol/L or higher or with previously diagnosed type 2 diabetes.

### 2.4. Determination of Chronic Kidney Disease

The estimated glomerular filtration rate (eGFR), an indicator of kidney function, was estimated using a formula from the Modification of Diet Renal Disease (MDRD) study: GFR (mL/min/1.73 m^2^) = 186.3 × (serum Cr) − 1.154 × (age) − 0.203 × (0.742 if women) × (1.21 if African-Americans) [22]. Reduced renal function was defined as an eGFR of less than 60 mL/min per 1.73 m^2^. For practical purposes, albuminuria was defined as a spot urinary albumin-to-creatinine ratio higher than 30 mg/g. CKD was defined as an eGFR of less than 60 mL/min per 1.73 m^2^ or albuminuria.

### 2.5. Socioeconomic Factors

The socioeconomic factors used in this study was defined on educational attained status. Education status was classified into two categories: (1) 0 years of schooling, primary school or junior middle school; (3) high school or above.

### 2.6. Health Behavior Factors

Alcohol consumption was evaluated based on the frequency of alcohol intake as recorded on the health interview questionnaire. We divided the residents into four groups: (1) No drinking; (2) less than once a week: (3) 1–4 times a week; (4) more than 5 times a week; (5) former but no present drinking.

For physical exercise, the participants were divided into two groups: (1) no physical activity; (2) physically active. For smoking, they were divided into three groups: (1) former smokers, quitted smoking prior to the survey; (2) current smokers; (3) non-smoking.

## 3. Statistical Analysis

Data were analyzed using Stata (version 11). Continuous variables were shown as mean ± standard deviation if they had normal distribution. Median and interquartile range were used to show skewed distributed continuous variables. The categorical variables were presented as absolute and relative (%) values or proportion. A two-tailed *p* value <0.05 was considered significant.

All participants were divided into 3 subgroups: Group 1, 40 years old ≤ Age < 50 years old; Group 2, 50 years old ≤ Age < 60 years old; Group 3, 60 years old ≤ Age ≤ 65 years old. Baseline characteristics within each subgroups were examined using the chi-squared test for categorical variables and Student’s t test or Wilcoxon rank-sum test for continuous variables.

The association of MetS with CKD was examined using logistic regression models. The first model was unadjusted. Next this model was expanded by adding covariates to adjust for factors that are likely potential confounders and unlikely to be in the causal pathway between MetS and CKD. These variables were age, history of stroke, history of coronary heart disease, smoking status, alcohol use, physical inactivity, education level.

The association of CKD and individual component of MetS were also examimed separately. The first logistic regression model was unadjusted, and the second model was adjusted for age, history of stroke, history of coronary heart disease, smoking status, alcohol use, physical inactivity, education level. All skewed distributed continuous variables were logarithmically transformed to normally distributed variables.

## 4. Result

Initially there were 763 participants in our community study, but 78 participants were excluded because of missing data for serum creatinine, serum HDL, serum LDL, serum fast glucose, anthropometric indexes, or ACR. Finally, 685 participants were included in the study. Among the total participants, 277 participants belonged to Group 1, 256 participants belonged to Group 2, and 152 participants belonged to Group 3.

## 5. Baseline Characteristics of Each Subgroups: (Table 1)

As shown in Table 1, the prevalence of history of coronary heart disease, history of hypertension, history of diabetes mellitus, abdominal obesity, elevated fasting glucose level, elevated blood pressure, CKD (*p* < 0.001) and elevated triglyceride level (*p* = 0.001) was significantly higher in Group 3 than in Group 2; urinary albumin-to-creatinine ratio, fasting glucose, serum triglyceride, and serum low density lipoprotein (*p* < 0.001), HOMA-index and serum uric acid (*p* = 0.002) were significantly higher in Group 3 than in Group 2. with the years old increasing, HOMA-index and serum uric acid were significantly higher (*p* = 0.002); with the years old increasing, eGFR was significantly lower in Group 3 than in Group 2 (*p* < 0.001).

**Table 1:** Baseline characteristics of perimenopausal women.

	Group 1 40 ≤ Age < 50 n = 277	Group 2 50 ≤ Age < 60 n = 256	Group 3 60 ≤ Age ≤ 65 n = 152	*p* value
**Clinical Characteristics**				
Age (Years)	44.87 ± 2.97	54.53 ± 2.30	62.44 ± 1.63	<0.001
Body Mass Index (kg/m^2^)	22.81 ± 3.12	23.67 ± 3.43	23.40 ± 3.27	0.30
Waist circumference (cm)	78.67 ± 8.45	82.63 ± 9.10	83.33 ± 9.25	0.34
History of diabetes mellitus (%)	2 (0.72)	17 (6.64)	17 (11.18)	<0.001
History of hypertension (%)	17 (6.64)	44 (17.19)	50 (32.89)	<0.001
History of coronary heartdisease (%)	2 (0.72)	3 (1.17)	9 (5.92)	<0.001
Current smoker (%)	2 (0.72)	2 (0.78)	0 (0)	0.69
Current alcohol use (%)	1 (0.36)	2 (0.78)	0 (0)	0.60
High school or above (%)	105 (37.91)	84 (32.81)	29 (19.08)	<0.001
Physical inactivity (%)	175 (63.18)	136 (53.13)	59 (38.82)	<0.001
Systolic blood pressure (mm Hg)	120.63 ± 16.50	129.48 ± 18.71	134 ± 19.02	0.06
Diastolic blood pressure (mm Hg)	76.67 ± 11.05	79.53 ± 10.37	79.82 ± 9.91	0.29
**Laboratory values**				
Serum creatitine (umol/L)	62.04 ± 9.37	64.61 ± 8.93	66.80 ± 10.05	0.26
Serum uric acid (umol/L)	288.93 ± 69.92	327.31 ± 77.31	336.24 ± 89.93	0.002
eGFR (mL/min/1.73 m^2^)	98.80 ± 18.02	90.13 ± 14.47	84.63 ± 14.22	<0.001
Urinary albumin-to-creatinine ratio (mg/g)	8.13 (6.01–12.02)	9.19 (6.59–13.26)	11.14 (7.74–17.28)	<0.001
Fasting glucose (mmol/L)	4.75 ± 0.65	5.03 ± 1.06	5.13 ± 0.93	<0.001
Serum C-reactive protein (mg/L)	0.77 (0.37–1.81)	1.29 (0.61–2.62)	0.88 (0.50–2.39)	<0.001
Serum triglyceride (mmol/L)	1.02 (0.75–1.38)	1.23 (0.91–1.62)	1.33 (0.98–1.8)	<0.001
Serum low density lipoprotein (mmol/L)	3.02 ±0.80	3.42 ± 0.89	3.40 ± 0.91	<0.001
Serum high density lipoprotein (mmol/L)	1.60 ± 0.31	1.60 ± 0.33	1.60 ± 0.32	0.52
HOMA-index (uU/mL)	1.61 (1.16–2.47)	1.81 (1.23–2.69)	2.18 (1.40–2.83)	0.002
**Metabolic disturbances and chronic conditions**				
Abdominal obesity (%)	122 (44.04)	160 (62.50)	102 (67.11)	<0.001
Elevated blood pressure (%)	88 (31.77)	140 (54.69)	108 (71.05)	<0.001
Elevated fasting glucose level (%)	17 (6.14)	45 (17.58)	33 (21.71)	<0.001
Low high densitylipoprotein (%)	43 (15.52)	36 (14.06)	26 (17.11)	0.71
Elevated triglyceride level (%)	42 (15.16)	58 (22.66)	42 (27.63)	0.006
Chronic kidney disease (%)	24 (8.66)	18 (7.03)	23 (15.13)	<0.001
Urinary albumin-to-creatinine ratio ≥ 30 mg/g (%)	23 (8.30)	16 (6.25)	18 (11.84)	0.142

Mean ± SD or median (25th to 75th percentiles) for continuous variables and absolute and relative (%) values for category variables are presented. HOMA-IR: Homeostatic model assessment of insulin resistance; Elevated blood pressure, elevated fasting glucose level, low high density lipoprotein cholesterol, elevated triglyceride level and metabolic syndrome diagnostic criteria were defined according to IDF.

## 6. Associations of MetS with CKD in Multivariate Logistic Model (Table 2)

As shown in Table 2, MetS was significantly associated with CKD with unadjusted Odds Ratio: 3.04 (1.79–5.14), *p* < 0.001.

**Table 2:** Associations of MetS with CKD in multivariate logistic model.

	Model One ^a^		Model Two ^b^	
	Odds Ratio (95% CI)	*p* Value	Odds Ratio (95% CI)	*p* Value
Total n = 685	3.04 (1.79–5.14)	<0.001	2.66 (1.54–4.59)	<0.001
Group 1 n = 277	2.84 (1.09–7.39)	0.032	2.10 (0.75–5.87)	0.159
Group 2 n = 256	5.62 (2.08–15.22)	0.001	6.79 (2.30–20.09)	0.001
Group 3 n = 152	1.65 (0.68–4.04)	0.27	1.73 (0.70–4.30)	0.24

^a^ Unadjusted; ^b^ Adjusted for age, history of stroke, history of coronary heart disease, smoking status, alcohol use, physical inactivity, education attainment.

After adjusting for age, history of stroke, history of coronary heart disease, smoking status, alcohol use, physical inactivity and education level, there was still strong association between MetS and CKD (muti-adjusted Odds ratio: 2.66 (1.54–4.59), *p* < 0.001). Although the adjusted relationship between MetS and CKD was not significant in Group 1 and Group 3, the relationship holds for Group 2 (muti-adjusted Odds ratio: 6.79 (2.30–20.09), *p* < 0.001).

## 7. Associations of MetS Components with CKD in Group 1 (Table 3)

As shown in Table 3, there was no association between MetS components and CKD in Group 1 (*p* > 0.05).

**Table 3:** Associations of MetS components with CKD in Group 1.

	Model One ^a^		Model Two ^b^	
	Odds Ratio (95% CI)	*p* Value	Odds Ratio (95% CI)	*p* Value
Abdominal obesity	1.56 (0.67–3.62)	0.30	1.20 (0.49–2.93)	0.68
Elevated fasting glucose	3.69 (1.10–12.38)	0.03	3.15 (0.88–11.34)	0.08
Elevated blood pressure	2.33 (1.00–5.42)	0.050	1.16 (0.16–3.23)	0.78
Low HDL cholesterol	2.48 (0.96–6.41)	0.06	2.47 (0.92–6.64)	0.07
Elevated triglycerides	1.54 (0.54–4.37)	0.42	1.43 (0.49–4.18)	0.51

^a^ Unadjusted; ^b^ Adjusted for age, history of stroke, history of coronary heart disease, smoking status, alcohol use, physical inactivity, education attainment.

## 8. Associations of MetS Components with CKD in Group 2 (Table 4)

As shown in Table 4, elevated blood pressure (unadjusted Odds ratio: 4.52 (1.28–16.02), *p* = 0.02) or Elevated fasting glucose (unadjusted Odds ratio: 3.69 (1.10–12.38), *p* = 0.03) was associated with CKD. However, after adjusting for age, history of stroke, history of coronary heart disease, smoking status, alcohol use, physical inactivity and education level, the relationship disappeared (*p* > 0.05).

**Table 4:** Associations between MetS components and CKD in Group 2.

	Model One ^a^		Model Two ^b^	
	Odds Ratio (95% CI)	*p* Value	Odds Ratio (95% CI)	*p* Value
Abdominal obesity	3.21 (0.90–11.38)	0.07	1.42 (0.51–4.00)	0.50
Elevated fasting glucose	3.69 (1.10–12.38)	0.03	2.2 (0.76–6.61)	0.15
Elevated blood pressure	4.52 (1.28–16.02)	0.02	1.16 (0.42–3.23)	0.78
Low HDL cholesterol	1.14 (0.57–2.26)	0.71	0.73 (0.31–1.67)	0.45
Elevated triglycerides	1.34 (0.46–3.94)	0.59	1.67 (0.55–5.13)	0.37

^a^ Unadjusted; ^b^ Adjusted for age, history of stroke, history of coronary heart disease, smoking status, alcohol use, physical inactivity, education attainment.

## 9. Discussion

Our present study established a positive and significant relationship between MetS and CKD in perimenopausal women aged from 50 or older to 60. Our data clearly illustrates that this relationship is independent of age and other potential risk factors of CKD. This includes a history of the following; coronary heart disease, smoking status, alcohol use, physical inactivity and education level. The value of our results lies in the fact that MetS is highly prevalent worldwide, with a prevalence rate ranging from 10% to 40% in divergent populations [23,24]. And intensive educational programs with a high-risk approach could improve the population awareness for screening and control of the CVD risk factors [25]. Diagnosing MetS in its earlier stages may contribute to slowing down the process of CKD as well as improving the patient’s quality of life. In turn, decreasing the economic burden on society and alleviating the suffering of those afflicted with CKD.

The association between MetS and CKD was recently analysed [26,27]. Chen *et al*. [26] established that MetS was an independent risk factor of CKD. They examined the association of MetS coupled with the risk of developing CKD in more than 6,000 subjects who participated in the Third National Health and Nutrition Examination Survey (NHANES III). Per their analysis MetS was found to be independently associated with an increased risk of CKD. A sizeable study utilising data from the Atherosclerosis Risk in Communities (ARICs) cohort confirmed a step-wise increase with the risk of developing CKD, per the MetS criteria met by nondiabetic adults, even after putting in place prophylactic measures for the possible development of diabetes mellitus (DM) and hypertension [28]. In 2007, Tozawa *et al*. [13] conducted a prospective study to examine metabolic syndrome as a risk factor for CKD in an Asian population.Six thousand three hundred and seventy one subjects (6,371) without CKD or diabetes mellitus at baseline from 1997 through 2002 in Okinawa, Japan were examined in this study for CKD. During the 5-year follow up exercise, 369 (5.7%) of the participants developed CKD. After adjusting for age, sex, current cigarette smoking, and alcohol drinking habits at baseline, the relative risk of developing CKD was determined to be 1.86 (95% confidence interval: 1.43–2.41, *p* < 0.0001) in subjects with MetS. Based on exhaustive studies conducted by Zhang *et al*. [29] on 2,310 Chinese study participants, aged 40 years and older, MetS was found to be significantly associated with CKD (including lowered eGFR and microalbuminuria) in Northern China. To the best of our knowledge, this study which was designed to examine the association between MetS and CKD in perimenopausal women population and trace the similarity of risk associations in spite of environmental and life style differences, is the first of its kind. Subsequently, our present study demonstrates the significant association between MetS and the risk of CKD in perimenopausal women aged from 50 or older to 60.

In addition, we also examined the relationship between components of MetS and CKD in perimenopausal women population. No association was found between these variables. Specifically referring to perimenopausal women aged 40 to 50 and those aged 60 to 65. Elevated blood pressure and high levels of fasting glucose were found to be significantly and positively associated with CKD. This association is dependent on other potential chronic kidney disease risk factors, which include coronary heart disease, smoking status, alcohol use, physical inactivity and education level. No suitable explaination could account for the varied relationships exhibited between the components of MetS and CKD; especially with regards to their different subgroups. Perhaps this may be attributed to fluctuating endocrine levels or the varied sex hormone levels of the different subgroups [8]. The cause behind this mechanism needs to be researched further.

Limitations of our present study should be noted. Firstly, its cross-sectional design makes it difficult to infer causality between MS and the risk of incurring CKD. Secondly, in our analysis we employed the use of eGFR as opposed to directly measuring GFR to define CKD. This process requires that serum creatinine (Scr) be calibrated by indirect method per US population data. The relationship between Scr and GFR varies with race, and of particular note is that the modification of diet in the renal disease formula was not validated for study of this Chinese population. Thirdly, we chose not to examine levels of sex hormone, thus we can only speculate on the impact sex hormone has on the relationship between MetS (it’s components) and CKD in perimenopausal women. Fourth, due to the limited information in our data, we could not analyze the effects of pharmaceutical drug used, such as non-steroidal anti-inflammatory drug which may have exacerbated the renal disease. Finally, only 685 participants were recruited in this study, a sample size that is relatively small.

## 10. Conclusions

In summary, our study explored the association between MetS and CKD in perimenopausal women. We found a significant relationship only for community perimenopausal women aged from 50 or older to 60. Further studies are warranted to elucidate the causal relationship between the metabolic syndrome and CKD in perimenopausal women aged from 50 or older to 60. The possible impact of sex hormones on the relationship between MetS (it’s components) and CKD in perimenopausal women, likewise warrants further investigation.
